# Digital sketch maps and eye tracking statistics as instruments to obtain insights into spatial cognition

**DOI:** 10.16910/jemr.11.3.4

**Published:** 2018-06-15

**Authors:** Merve Keskin, Kristien Ooms, Ahmet Ozgur Dogru, Philippe De Maeyer

**Affiliations:** Ghent University, Ghent, Belgium; Istanbul Technical University, Istanbul, Turkey

**Keywords:** sketch map, eye tracking, cognitive cartography, spatial cognition, usability, individual differences, region of interest

## Abstract

This paper explores map users' cognitive processes in learning, acquiring and remembering information presented via screen maps. In this context, we conducted a mixed-methods user experiment employing digital sketch maps and eye tracking. On the one hand, the performance of the participants was assessed based on the order with which the objects were drawn and the influence of visual variables (e.g. presence & location, size, shape, color). On the other hand, trial durations and eye tracking statistics such as average duration of fixations, and number of fixations per seconds were compared. Moreover, selected AoIs (Area of Interests) were explored to gain a deeper insight on visual behavior of map users. Depending on the normality of the data, we used either two-way ANOVA or Mann-Whitney U test to inspect the significance of the results. Based on the evaluation of the drawing order, we observed that experts and males drew roads first whereas; novices and females focused more on hydrographic object. According to the assessment of drawn elements, no significant differences emerged between neither experts and novices, nor females and males for the retrieval of spatial information presented on 2D maps with a simple design and content. The differences in trial durations between novices and experts were not statistically significant while both studying and drawing. Similarly, no significant difference occurred between female and male participants for either studying or drawing. Eye tracking metrics also supported these findings. For average duration of fixation, there was found no significant difference between experts and novices, as well as between females and males. Similarly, no significant differences were found for the mean number of fixation.

## Introduction

Maps convey direct and indirect information about the objects they represent. In addition to information about the location, name, shape, and size of objects, maps provide spatial relationships among these objects. When a person needs to find a geographic phenomenon, select a route, navigate, or estimate a distance, (s)he tends to memorize the relevant direct or indirect information on a map. Together with human (perceptual, cognitive, and visual) abilities, the retrieval of spatial information is strongly correlated with map learning. Map learning is distinguished from other learning concepts because (i) it requires comprehending and memorizing the direct information presented in maps and (ii) all the information to be learned is presented at once. These two characteristics of map learning allow map users flexibility regarding when, how and in which order they execute tasks such as selecting and focusing [ 1 ]. Hence, each user/user group develops different strategies for approaching the spatial information on maps (e.g. [ 2, 3, 4, 5, 6 ]).

This paper intends to examine map users’ cognitive processes of learning, acquiring and remembering information presented via screen maps. The map users targeted in the paper are broadly categorized as novices and experts considering their individual group differences of age, gender, ethnicity and language. The main research question addressed in this paper is “do novices and experts use different strategies while studying maps and recalling maprelated information?”. In this context, the experiments are designed based on the principles and strategies defined by Thorndyke & Stasz [ 1 ], Montello et al [ 7 ] and Ooms et al [ 6 ]. Various methods (e.g. think-aloud, eye tracking, interview) have been applied to evaluate the recall of map-related information from memory (e.g. [ 8, 9, 10, 11 ]). Sketch maps are one of these methods, since they concretize the extracted information from a cognitive map (also called a mental image, map image, mental map) through drawing. This concept is further discussed in the literature review in the following section.

User testing methods can be mixed for many reasons such as to enrich the quantitative research in cartography, to better contextualize map design and use/user recommendations, to improve the consistency and detail of results, and to adopt and adapt new approaches to our study design [ 9, 12, 13 ]. In our study, we also use mixed methods of sketch maps, eye tracking (ET) and a post-test questionnaire. Both eye tracking and sketch map methods individually provide a considerable amount of valuable information related to map users. Therefore, the combination of these methods potentially brings advantages to the user study design in terms of methods, materials or user needs and to the evaluation of results, in addition to yielding additional insights about map users’ behaviors. In fact, sketch maps and ET can be considered as complementary to one another; for instance, ET metrics can explain an outcome obtained from sketch maps or vice versa. ET is also valuable for the validation of results acquired from one method with those from the other.

## Literature Review

### Map Learning and cognitive map production

Learning and remembering cartographic information are associated with how the human cognitive system addresses geographic information presented via maps to produce cognitive maps. Especially in the past four decades, cognitive maps have become an intriguing research topic in geography [ 14, 15 ] as well as in neuroscience [ 16, 17, 18 ] and psychology [ 19 ]. The discovery of place cells [ 18 ] and grid cells [ 20 ] stands as evidence that there exists a group of neurons in the brain that are responsible for our cognitive maps and inner navigation. Various studies in cartography have emphasized how we see maps and how we derive meaning from them (e.g. [ 21, 22, 23 ]).

Learning a map involves two interacting cognitive factors: (i) control processes and (ii) the memorial system [ 24 ]. The first cognitive factor of map learning refers to matching the map to the prior knowledge existing in the memory and the achievement of the map-learning task. In this respect, prior knowledge can originate from general and specific map knowledge. General map knowledge helps in distinguishing maps from other spatial displays. It enables the encoding of maps and the development of strategies for map learning.

The influence of general map knowledge on map learning depends on the perception of “maplikeness” and the degree of expertise [ 25, 26 ]. Past studies have presented that map learning is more efficient when the stimulus is more maplike [ 27, 28 ] and that experts and novices differ somewhat in terms of their ability to learn and remember information presented via maps [ 1 ]. If an effective spatial behavior requires using vector-like information about distances and directions, this information should be stored as maplike representations [ 19 ]. O’Keefe and Nadel [ 18 ] proposed that the spatial learning system forms cognitive maps through exploration and a later study claimed that associative learning integrates “all kinds of spatial information spontaneously into a unitary maplike representation” [ 19 , p. 288]. In addition, Portugali [ 15 ] listed much research showing that without any prior training, children can comprehend aerial photographs at appropriate scale and are able to use them as maps. This outcome proves that maplike behavior is very fundamental in human development and that mapping skills develop much earlier than predicted.

Expertise plays a role equally important to maplikeness in map learning. To recall the locations and configurations of spatial objects from the memory usually requires experience with cartographic products in which topographic and topological information are represented by graphic symbols [ 29 ]. Unlike general map knowledge, specific map knowledge stems from the modifications of related information in the long-term memory (LTM) based on the degree of familiarity with particular map representations. These representations are called knowledge-weighted cognitive maps, which are constructed from perceptual stimulus, initial map-learning conditions and the way that the information has been used [ 30 ].

The second cognitive factor of map learning - the memorial system - addresses the mode of representation and the resources to store and maintain cognitive maps [ 24 ]. A map image holds both features represented by visual variables and structural information. The structural information refers to a spatial framework such as geometric and metric relations among features, whereas the visual variables, described by Bertin [ 31 ] are the fundamental units that help distinguishing map symbols and encoding information presented via maps. Visual variables (i.e. position, size, shape, value, color hue, orientation, and texture) play a key role in cartographic design because their use for map symbols has a great impact on visual attention and perception. How these variables are perceived depends on their property (i.e. selective, associative, ordered or quantitative) (for further reading see also [ 32 ]).

As Kulhavy & Stock [ 24 ] argued, we should understand whether our cognitive map is just a collection of features and their properties or it encodes structural relationships as well. The answer depends on the similarity of the map and its cognitive map. Clearly, all individuals create their own unique cognitive maps. Cognitive map creation occurs in a fashion similar to Haken’s [ 33 ] theory of information and self-organization (synergetics). Synergetics, originating in physics, is a method and a philosophy to explain the formation and the self-organization of individual elements in an open and complex system for the stability and the survival of the whole system [ 34 ]. Let us try to explain cognitive map creation in the human brain, which is also an open and complex system. When the brain receives spatial information through the external world (a physical environment or maps), the cognitive system constructs a cognitive map out of a partial set of features stored in the brain as internal representations. During this procedure, the cognitive system is governed by order parameters, which are the common principles shaped by the interactions among the individual elements of the system [ 35 ]. According to the synergetics theory, atoms form order parameters, and order parameters enslave (govern) atoms [ 34 ]. Therefore, cognitive map construction can only be achieved “when a certain mapping principle, or mapping order parameter, enslaves the various features” through associative memory [35, p. 14]. As a result, the cognitive map is successfully created from the interaction of the internal and external representations of the environment influenced by order parameters (for further reading, see [ 36, 37 ]).

**Figure 1.**
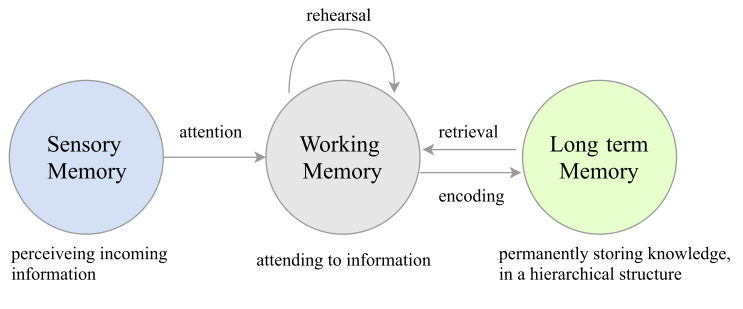
The interaction between memory type

Nevertheless, our cognitive system is capacity-limited in terms of encoding new information for storage in LTM and also of retrieving and making use of old information already in memory [ 24 ]. As Atkinson & Shiffrin [ 38 ] proposed, memory involves a sequence of three stages; sensory memory, working (short-term) memory, and LTM. Sensory memory holds the information gathered through all our senses for a brief time span and then decays and is lost. A part of the information in the sensory memory is transferred to the working memory (WM). The WM can receive selected inputs from the sensory register, as well as from LTM. WM is active during encoding and storing new information for short time periods or during the retrieval and use of the old information. On the other hand, LTM retains the informative knowledge (memories, things we learn, etc.) permanently, because it has an almost limitless capacity. Once WM transfers information to LTM, this information can be remembered for longer periods. This transmission is called the learning process and requires rehearsal [ 6, 24, 38 ] (Figure 1). Unlike LTM, WM has a limited capacity in terms of individual items of information called chunks. A chunk is any stimulus that has become familiar, hence recognizable, through experience [ 39 40 ]. To be able to draw cognitive maps, the chunks of information obtained from maps must be transferred from LTM to WM.

Besides WM capacity, object-location memory and landmarks play principal roles for the cognition of the spatial objects, the formation of the cognitive representations and the recall processes of those. According to Tversky [ 41 ], the brain reorganizes the information entirely through (i) hierarchical organization or categorization, (ii) the use of perspective, and (iii) the use of landmarks or cognitive reference points. Once people learn the locations of objects, they can establish the spatial structure of a map to form a mental representation or cognitive map of the environment. Cognitive maps hold information not only about spatial objects, but also the relations and distances between objects, even the absence of spatial objects. The distortions in the spatial object positions and their relations are indicators of hierarchical encoding and perceptual organization [ 42 ]. In this context, landmarks and routes are considered as the core units of a spatial representation and are helpful primarily for orientation [ 43, 44 ].

In cartography, empirical studies focusing on map design and spatial cognition are increasing, however, only a number of them devoted to the exploration of cartographic elements (e.g. visual variables) which play an important role in cognitive map formation (e.g. [ 29, 42, 45 ]). Hence, we cannot yet formulate the cognitive map construction precisely and the assessment of this procedure is not straightforward. Nevertheless, sketch maps, considering their complexity, can be utilized as one of the sources to examine this process.

### Sketch maps

Sketch maps are the reflections of individual cognitive maps. According to Forbus, Usher & Chapman [ 46 ], sketch maps are defined as “compact spatial representations that express the key spatial features of a situation for the task at hand, abstracting away the mass of details that would otherwise obscure the relevant aspects” (p. 61). Therefore, the interpretation of sketch maps reveals the underlying task-related cognitive process of individuals. A sketch map is also a three-dimensional representation through space, time and sequence because the ordered retrieval of movements within time and space results in our cognitive maps [ 47 ]. The hierarchical order of nodes and paths drawn on the sketch maps represents the hierarchical order of information (primary-level, secondary-level, and so on) presented on the maps. As Lynch [ 48 , p. 86] describes, “the sequence in which sketch maps were drawn seemed to indicate that the image develops, or grows, in different ways.” The earlier the element is recalled, the more important it is to a person. Lower hierarchical levels correspond to decreasing amounts of spatial information, decreasing frequency of use and greater difficulty of remembering [ 49 ]. Hence, drawing order can yield insights into how these elements are stored in the user’s memory. In other words, if an element is drawn earlier, it means that it is more accessible in LTM, thus, retrieved with ease [ 6 ].

Sketch maps have been used in several research projects as a data collection method to investigate the cognitive processes of map users (e.g. [ 46, 47, 50, 51, 52 ]). Sketch maps are often combined with the think aloud procedure as a complementary data collection method (e.g. [ 6, 53 ]) because thinking aloud gives insights into the user’s unfiltered thoughts. Thinking aloud itself, however, has the disadvantage that it also consumes part of the user’s memory capacity.

### Retrieving a sketch map from memory

Spatial memory is controlled by perception-based and memory-based processes [ 42 ]. Sketch maps underlie the map users’ cognitive procedures of learning and remembering the information presented via maps. Hence, it is essential to identify the cognitive procedures involved during both learning and the retrieval of map-related information. Learning requires to create a higher framework of specific graphic features (e.g. map-inherent features or grids). While studying, a map reader first perceptually divides the map into a number of spatial chunks. In this context, the structuring map elements, such as roads, hydrographic features or gridlines, initiates chunking process, thus, helps regionalizing the map and assists learning of map elements and their spatial relations. These structuring elements represent the spatial information of the map content in a hierarchically structured fashion and form fundamental units of cognitive maps, therefore, facilitate the perception and recognition of object locations [ 42 ].

The first step of retrieval process is the orientation of the participant regarding the task (i.e. establishing a strategy to execute the task from the beginning to the end) and the surroundings (in this case, the drawing environment and its tools). The second step is task execution, in which participants form links between cognitive processes through WM and LTM. In chronological order, the participant first consults WM to check whether there is information about map elements that must be drawn. If the information exists in WM, the participant draws these elements; if not, he must consult LTM, which is responsible for the recalling act. For a participant to draw an element whose information is stored in LTM, this information needs to be transferred to WM. Afterwards, evaluation occurs for editing or redrawing, and then, the participant asks WM once again to finalize the procedure [ 6 ]. It is important to remember that this procedure is repetitive and continues until the participant is satisfied with the result. During this procedure, the sensory memory captures the image of the sketch map and transfers it to the WM. The memories of this original stimulus, which were previously stored in LTM, need to be recalled. Once the participant retrieves that information, (s)he can compare the sketch map with the original stimulus depending on the location, size, shape, color, etc. The retrieval process for chunks of information requires activation of the related information. This activation involves pointers, schemas and links between schemas stored in LTM. These pointers activate and retrieve the desired chunks of information from LTM and place them in WM [ 6 ].

### Eye tracking

It is known so far that the early beginnings of perceptual organization is evidenced by the first fixation on a visual stimulus [ 42 ]. The fixation-related behavior and other eye movement data can be measured via eye tracking which is a widely used quantitative user-testing method. Eye tracking has contributed to human-computer interaction usability studies in numerous disciplines varying from psychology to software engineering, marketing, sports, aviation, navigation and so forth (e.g. [ 3, 54, 55, 56, 57, 58, 59 ]). Many cartographers also employed eye tracking in their usability research, especially for the assessment of visual elements (e.g. [ 4, 10, 29, 52, 60, 61, 62 ]).

As explained earlier in the previous chapter, visual elements in topographic maps assist learning and recognition of location of map elements. Some eye tracking research has revealed how a map user processes those visual elements (e.g. [ 29, 44, 63 ]). Eye movement statistics, which can be linked to the cognitive processes when a participant interact with visual stimuli on the screen, consist of a list of pixel coordinates on the screen regarding various positions of the gaze (POR: point of regard). From the raw data, useful metrics such as how long (fixation duration) and how often (fixation count) a person focuses on a specific area of interest, together with his scan-path characteristics (the length and speed of the gaze activity), can be derived [ 61 ]. These metrics can also be analyzed for specified regions of the stimulus, called Areas of interest (AoIs). AoIs are subregions of a stimulus that are of high importance for a hypothesis and are created based on the semantic information of the stimulus [ 64 ].

Our literature study showed that there is a lack of research on the sources of individual differences (e.g. expertise, gender, etc.) and the relationship between the organization of spatial thinking and geographic space. Furthermore, there is a limited empirical evidence on user’s cognitive processes involved in map-related tasks, although cartographers hold theoretical knowledge about usability and design issues of maps. Therefore, this paper aims to evaluate the abovementioned cognitive process on a 2D static map to determine the cognitive abilities and/or limitations of map users when they first study the map and retrieve this information later. In this context, we propose collecting data via digital sketch maps, instead of conventional pen and paper method, to be able to link this with ET statistics. Both the ET data and the sketch maps give insights in the users’ cognitive processes, but from a different angle. By triangulating the obtained insight, a deeper understanding regarding individual differences of map users can be obtained.

## Methods

### Participants

A total of 56 participants took part in the study, with 24 experts and 30 novices. The numbers of female and male participants were 7 and 23, respectively, for novices and 13 and 11, respectively, for experts. The ages of 96% of the participants ranged between 18 and 34, which corresponds to a rather young user group. The novice participants were undergraduate Business and Economy students whose ages varied between 18 and 24 years and who gained credits in return for their participation. The expert group, whose ages ranged between 25 and 34, consisted of participants who had at least a MSc. in Geography, Geomatics Engineering or related areas, and all of them were affiliated with the Department of Geography (Ghent University). The majority of the participants were Belgian (native language=Dutch), and there were six Asian expert participants (native language=Chinese). The experiment itself was designed in English.

While experts work with cartographic products on a daily basis, novices use cartographic products from time to time (e.g. Google maps) and were not trained before the experiment. Eight female and eight male experts had participated in a user experiment with ET previously. Two novice males indicated that they had participated in a user study before. The remaining 38 participants were taking part in user testing for the first time. All participants unanimously indicated in the post-test questionnaire that the map stimulus was not familiar to them.

### Apparatus and recording

The experiment was conducted in the Eye Tracking Laboratory of the Marketing Department of Ghent University. The participants’ eye movements were recorded with an SMI RED250 eye tracker mounted to the stimulus monitor. The stimulus was shown on a 22” color monitor with 1680 x 1050 spatial resolution. We did not use a chin rest and the average distance between the participant and the monitor was 65 cm. Simultaneously with the gaze recording, we performed EEG (electroencephalogram) measurements to estimate the cognitive load. However, it is beyond the scope of this paper to attempt to explain theoretical background of EEG data acquisition, the synchronization of EEG and ET and related analysis.

### Materials

**Figure 2.**
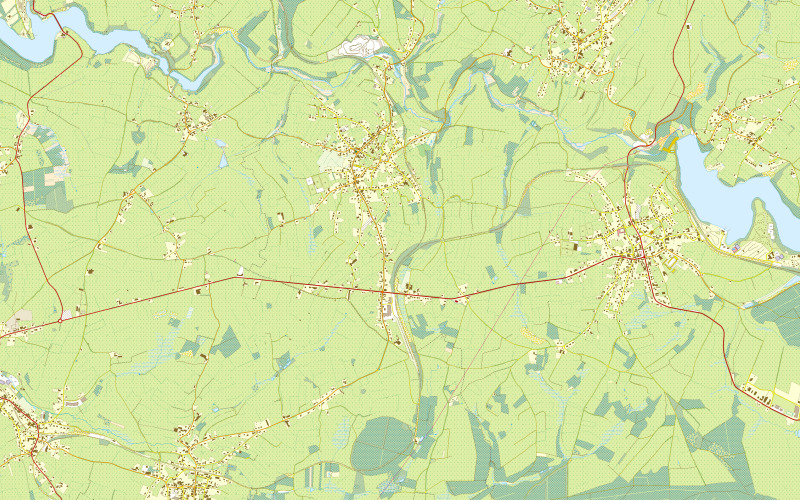
Original map stimulus shown in memory task (This map stimulus is the same material used by Ooms (52). This data was produced by Belgian national mapping agency, NGI/IGN (Nationaal Geografisch Instituut/Institut Géographique National)).

The stimulus was selected from the Belgian 1:10k topographic map series (Figure 2). We paid attention that it was not too complex yet contained some specific main structuring elements. To combat the learning effect, the selected map did not cover a well-known area/city.

### Procedure

Participants were instructed to study the map stimulus – for as long as they wanted – to be able to remember the main structural elements (rivers, roads, water bodies, etc.). Once they thought they had studied the map long enough, they pressed a certain key as instructed beforehand and thereby exited the first part of the assignment. Next, they had to draw this map from memory by using MS Paint. This tool was selected because neither experts nor novices would need any prior training. After the execution of the task – in other words, drawing the sketch map – participants used a special key to terminate the task. There was no time limitation for either the studying or the drawing part. While participants studied and drew the map, their eye movements were recorded.

### Sketch maps analysis

The first step of sketch map analysis was to quantify the information presented within the maps. Therefore, we determined the structural map elements on the original map/stimulus and then counted and classified them into four main categories: hydrology, land-cover, settlements, and roads. The original map consisted of four hydrographic features, four land-cover features, eight residential areas/settlements, and ten roads (in total, 26 map elements).

The sketch maps were analyzed based on the literature on cognitive processes and sketch map evaluation for cartographic usability (see previous section). In this context, two main criteria were identified; (i) drawing order and (ii) the score on drawn elements.

#### Drawing order

Drawing order information was derived from the registered eye tracking video and each participant’s data were processed individually. For the assessment of drawing order, the scoring system used by Ooms et al [ 6 ] was implemented. The scoring was 100, 50, 25, and 5 for the first, second, third, and fourth elements of a certain category, respectively. If a certain element did not exist on the sketch map, it did not receive any point. The rationale behind this scoring algorithm is simply assigning the highest score on the first drawn element and the least to the last drawn one. Among the first three classes (i.e. drawn elements), the weight is halved in value for each consecutive class so that the first drawn element stands out more. The last drawn element (i.e. fourth class) should have the least score, but not zero, since it is drawn on the sketch map. Therefore, its weight equals to the 1/5 of the third class. Finally, the average scores for each map category were calculated separately for expert and novice groups. Higher scores indicated that a certain element belonging to one of the four categories was drawn earlier. Therefore, 100 points would mean that all participants drew this category first. In this way, drawing order analyses contributed to the understanding of the hierarchical construction of the cognitive map.

The variables considered for the scoring of the drawn map elements were presence and accuracy (position), size, shape, and color, which corresponded to the qualitative characteristics of the sketch maps. The scoring provided information about how well the sketch map was executed (complete and accurate) and accordingly, how well the cognitive map was constructed.

#### Score on drawn elements

##### Presence and accuracy

The scoring system as used by Ooms et al. [ 6 ] was implemented to quantify the position of map features. If present and in the correct relative location, an object scored one point. If present and in a considerably wrong relative location, an object scored half a point. Finally, if absent, an object scored zero point. If a person successfully located every map element in the correct location, (s)he scored 26 points (total number of map elements). The results were expressed in percentages with 26 points representing 100%.

##### Shape, size and color

The shape, size and color characteristics of drawn elements were ranked by employing a system similar to that used by Billinghurst [ 50 ]. Their ranking scale was only modified to a 100-point scale; therefore, an incorrect score was 33.3, a partially correct score was 66.7, and a correct score was 100. Here, the participant’s drawing ability was neglected, and instead, we focused on how well the sketch map represented the area in the topographic map. For instance, linear objects such as roads and rivers should be illustrated as lines with varying thickness, and when individual roads connect, they should picture the overall road construction. Different logic should be followed for the aggregation of areal objects such that the individual buildings can be grouped and drawn as a single element (i.e. settlement), since the participants were particularly asked to draw the main structural elements. Additionally, only the major shape characteristics of the map elements were taken into consideration for scoring. For instance, both roads and railroads could be drawn as single lines, although they were depicted by double lines in the original map.

#### Aggregation presence & accuracy (1), shape (2), size(3) & color (4).

Presence & accuracy, shape, size and color of drawn elements show “how well” the sketch maps were drawn. Until this point, we have tried to evaluate the influence of each criterion individually. However, the aggregation of all criteria used for scoring the drawn elements can offer a more objective measure to compare the quality of sketch maps. Inherently, the quality of sketch maps reflects the performance of participants. We treated each of the four parameters as if they have equal importance for the overall performance of a participant, and thus, we assigned each parameter the same weight. Overall performance scores were calculated as the average of individual performances for the four different groups (expert females, expert males, novice females and novice males) in a 0-100 scoring scale.

### Eye tracking metrics

In addition to extracting the drawing order information from eye tracking data, eye tracking metrics such as the number of fixations per second and the average duration of fixation were analyzed. Similar to Ooms et al. [ 6 ], the number of fixations per second was considered instead of the fixation count because the fixation count is an absolute measure that is related to the length of the trial. Since every participant completes the task in a different time span, the fixation count would be merely a reflection of the trial duration. It is important to note that there is a strong relationship between the number of fixations per second and another widely used metric, average fixation duration. The longer the fixation durations are, the fewer the fixations per second. The fixation duration is also linked to the cognitive processes of the visual stimulus. Longer fixations may indicate that reading the map becomes harder, which causes a rise in the cognitive load [ 61, 65 ], or that the user finds the map or a certain part of it interesting [ 52 ]. People also concentrate their fixations on the most informative parts of the visual stimulus [ 66 ].

These metrics were further complemented with trial durations to study the map on one hand and to draw the associated sketch map on the other hand (results presented separately in 5.1). Although there was no time limitation for both study and drawing parts of the memory task, trial times give insight about motivation and top-down attention. Inherently, longer trial durations for studying the map indicate higher level of interest or difficulty in storing the information in memory.

Furthermore, some ET metrics were analyzed for specific AoIs. These were created on the basis of a previous study of Ooms et al. [ 5 ] which implemented the same stimuli. This study revealed that, based on a gridded approach of AoI, users tended to focus most on main structuring topographic characteristics in the map stimulus (i.e. major roads, settlements and hydrographic features). In this study we thus selected the same object to be included in the AoI. Buffers were created around the linear features similar to what was done by Bargiota, Mitropoulos, Krassanakis & Nakos [ 67 ]. Based on the accuracy of eye tracker (0.5°) and the viewing distance (65 cm), buffer size was set to 21 pixels. In this context, the statistics such as how quickly participants notice an element (time to first fixation), how much time the participants spent in the region (dwell time), how many fixations occurred (fixation count, the number of fixations per second) and for how long (average fixation duration) were considered. These metrics were further complemented with trial durations to study the map, on the one hand, and to draw the associated sketch map on the other hand (results presented separately in 5.1).

## Results

### Trial durations

**Figure 3.**
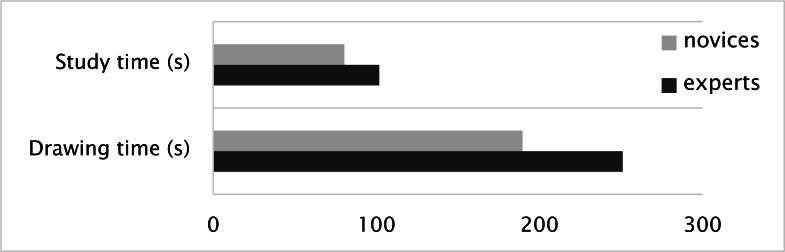
Trial durations of experts and novices.

Trial durations were assessed in two phases: (i) study time for the map stimulus and (ii) the drawing time for the sketch map. Figure 3 illustrates a general overview of the study and drawing performances of experts and novices. The graph clearly shows that drawing took approximately twice – or in some cases more than twice – as much time compared to the study phase.

#### Study time

**Figure 4.**
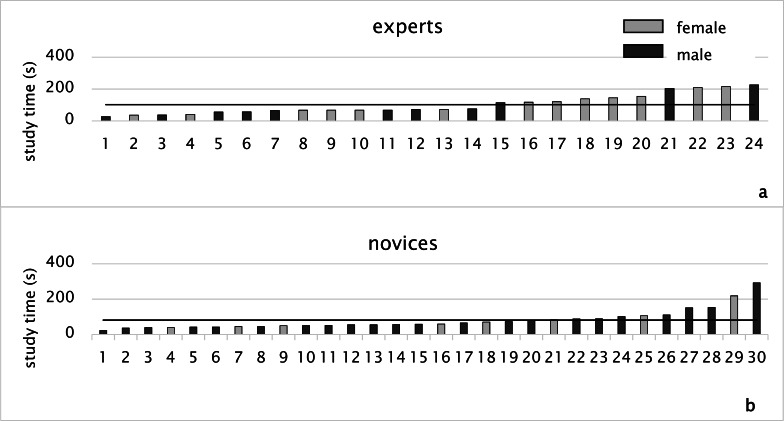
Study time of experts (a) and of novices (b) (black line: average)

The average (mean) time for studying the map was 102.7 s (N= 24, MED= 72.0 s, SD= 61.7 s) for experts with a minimum of 27.1 s and a maximum of 226.6 s (Figure 4a) and 81.5 s (N= 30, MED= 59.2 s, SD= 57.6 s) for novices with a minimum of 23.2 s and a maximum of 292.8 s (Figure 4b). If we classify the performances of participants regarding to study time, 17% of experts spent 0-50 s; 41%, 50-100 s; 21%, 100-150 s; and 21%, 150 s and more. On the other hand, 35% of novices spent 0-50 s; 46%, 50-100 s; 6%, 100-150 s; and 4%, 150 s and more. The results confirm that experts allocated more time in studying than novices did.

#### Drawing time

**Figure 5.**
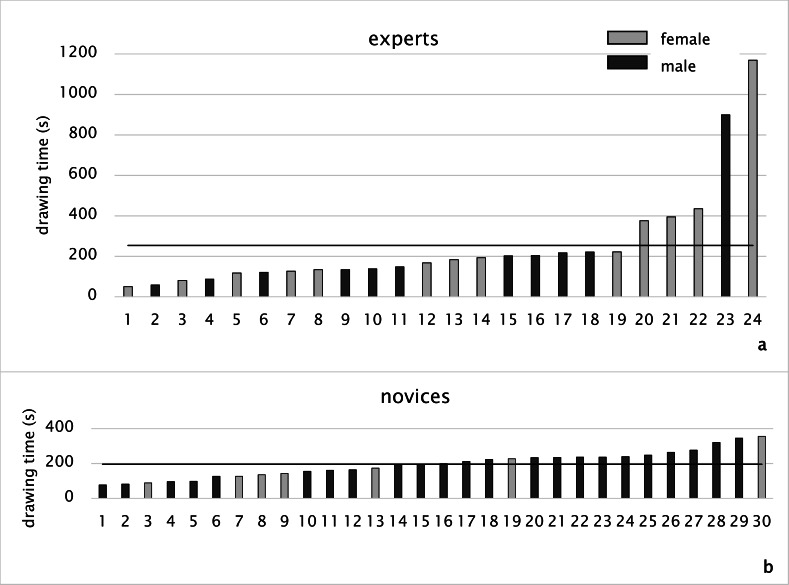
Drawing time of experts (a) and of novices (b) (black line: average)

As for the study part of the memory task, there was no time limitation for the drawing part. The average drawing time for experts was 253.5 s (N= 24, MED= 175.3 s, SD= 262.9 s) with a minimum of 76.5 s and a maximum of 356.1 s (Figure 5a), whereas the average drawing time was 195.4 s (N= 30, MED= 196.9 s, SD= 75.6 s) for novices with a minimum of 50.2 s and a maximum of 1169.4 s (Figure 5b).

The time spent on sketching the map might correspond to the richness of detail depicted in the sketch map, the difficulties encountered due to the lack of experience (e.g. unfamiliarity of the task and of the drawing tool), or recall issues. The fact that novices were faster in both studying and drawing may explain that novices were not aware of procedures involved in map production, did not exactly know what to remember. In addition, they are less involved with cartography, thus they might have paid less attention to having good results. Since the average drawing time for experts is greater than that for novices, some experts spent the longest time on the task. The extreme values that occurred in the expert group can be explained by the richness of main structural elements on the sketch maps. These sketch maps were detailed, contained larger numbers of structural elements and scored higher than the average among their group. Unlike in the expert group, there was a more balanced trend among novices (Figure 5b). However, the novices who spent the longest time (corresponding to one-third of the time that experts spent) received scores equal to those for experts on their sketch maps.

A Kolmogrov-Smirnov test was used to test of normality on the dependent variables, which are study time and drawing time. For both data, p= 0.000 suggested strong evidence of the data was not normally distributed (D_study_(54) = 0.209, p < 0.05, and D_drawing_(54) = 0.258, p < 0.05). Since the data did not fit normal distribution, Mann-Whitney U non-parametric method was chosen to test significance of the results. It can be concluded that the differences occurred between novices and experts while both studying (M= 90.9 s, SD= 59.9 s) and drawing (M= 221.2 s, SD= 194.3 s) were not statistically significant (U_study_ = 275, p= 0.139 and U_drawing_= 320, p= 0.486). Similarly, no significant difference emerged between female and male participants for either studying or drawing (U_study_ = 265, p =0.179 and U_drawing_= 321, p= 0.734).

### Sketch Map Analysis

#### Drawing Order

Although the spatial distributions of elements on the sketch maps were not properly structured or were even distorted, the drawing order (sequence) was similar to that found by Lynch [ 48 ].

**Figure 6.**
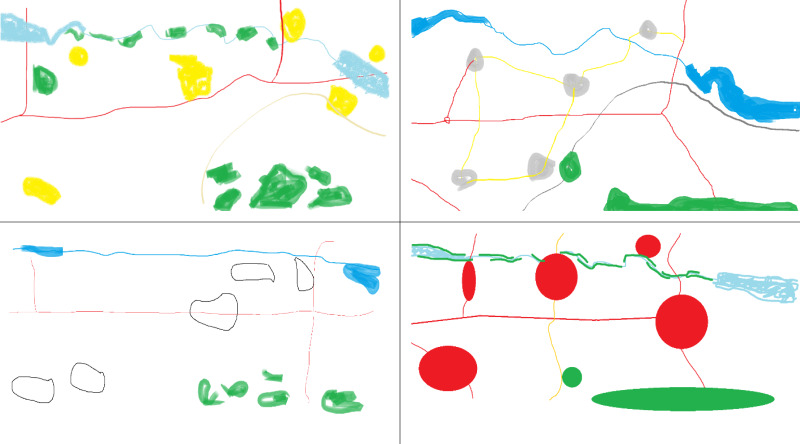
Sketch map examples (top and bottom left: novices, top and bottom right: experts)

**Figure 7.**
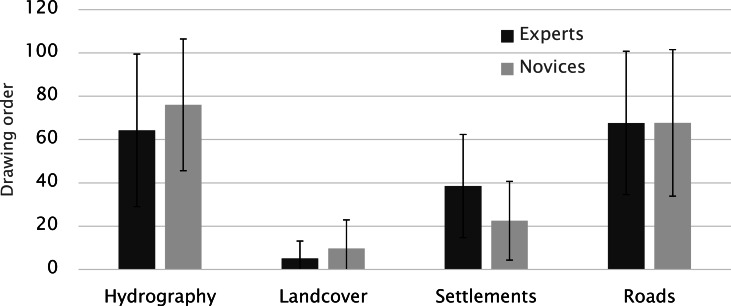
Scores for drawing order (Error bars indicate SD).

Figure 6 depicts the examples of sketch maps drawn by experts and novices for the memory task. According to the average scoring results of all participants, the hydrography (M= 70.1, MED= 50, SD= 32.5) and road (M= 67.7, MED= 50, SD= 33.6) categories were linked to the highest scores, whereas settlements (M= 30.5, MED= 25, SD= 21.8), and land-cover (M= 9.1, MED= 5.0, SD= 11.7) were associated with the lowest ones (Figure 7). This result means that the majority of participants drew hydrographic objects first. The drawing orders for experts and novices show a slight difference. While experts drew roads first, novices focused more on hydrographic objects such as rivers and water bodies. Hydrography and roads form the main structural elements on the maps. Settlements and land-cover elements (in this case, forest) were drawn third and fourth, respectively, for both user groups.

The fact that both experts and novices drew linear objects (hydrography and roads) first can be explained by the hierarchical structures of schemas in LTM. This fact gives a clear idea that the sketch maps are hierarchically constructed. This finding corresponds to what Huynh and Doherty [ 47 ], Huynh, Hall, Doherty & Smith [ 68 ] and Ooms et al. [ 6 ] found. They discovered that participants start drawing their sketch maps with the main linear structures and continue with other landmarks. Furthermore, female participants started with hydrographic objects, while male participants chose roads in the first place. Accordingly, both females and males drew settlements in the third place, and land-cover objects in the fourth place

#### Score on drawn elements

##### The presence and accuracy

**Figure 8.**
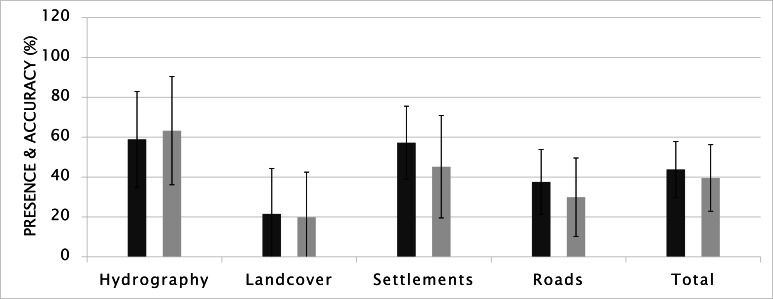
Presence and accuracy scores (Error bars indicate SD).

A Kolmogorov-Smirnov test was used to test for normality on presence and accuracy, D(54) = 0.090, p= 0.200 indicated that the data was normally distributed. Based on the average scores of all participants, the average location score was 41.3 (N=54, MED= 43.3, SD= 14.9). Experts placed map elements slightly more accurately than the novices did, but according to two-way ANOVA, no significant difference emerged, with F(1,55)= 0.888 and p= 0.350. The most pronounced performance difference between two groups occurred when placing the settlements (12.0%) (Figure 8). The reason for this finding could be explained by the amount, the complexity and the distribution of elements falling into this category. The original stimulus contained eight residential areas, which was the highest number of elements that a category held. Inherently, remembering all of them together with their positions would be harder, especially for novices, compared to other categories having fewer than eight elements. The more isolated the feature was, the more distinctive and easier to remember it became. Hence, the isolated settlements stood out more, and participants tended have higher probabilities of drawing them.

On the other hand, the presence and accuracy results favored females with a 6.3% difference. However, this difference was not statistically significant according to twoway ANOVA, F(1,55)= 1.672 and p= 0.101.

##### Shape, size and color

**Figure 9.**
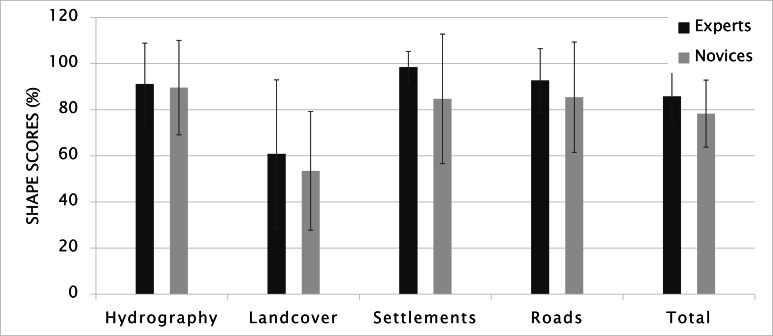
Shape scores (Error bars indicate standard deviation).

Based on average scores all participants, the average shape score was 82.1 (N=54, MED= 83.3, SD= 12.9). Figure 9 shows the shape scores for experts and novices based on the four main map element categories. A KolmogorovSmirnov test was used to test for normality on shape (D(54) = 0.131, p= 0.022), size (D(54) = 0.144, p= 0.007) and color (D(54) = 0.309, p= 0.000). The test results indicated that the data was not normally distributed. Experts illustrated the shape of the map elements 7.5% better than novices did, and Mann-Whitney U test showed that this difference was significant, with U_shape_= 247 and p= 0.044. Similar to the results for presence and location, the greatest difference in performances between novices and experts occurred in settlements at 13.8%. On the other hand, female participants outperformed males with a 5.9% difference which was not significant according to Mann-Whitney U test (U_shape_ = 249, p= 0.077).

**Figure 10.**
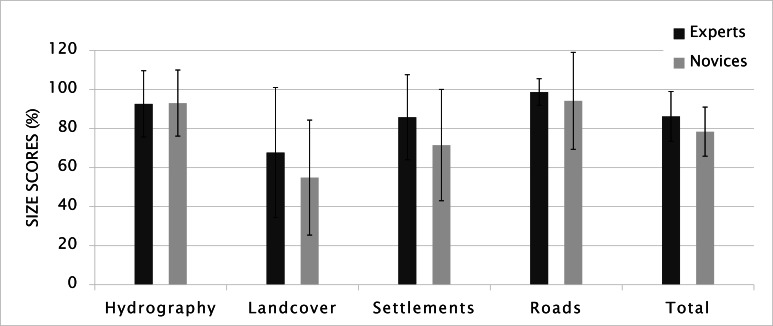
Size scores (Error bars indicate standard deviation).

Size is one of the most effective visual variables in terms of its selectiveness, associativity, and ease of perception as ordered. Larger elements can be perceived immediately compared to smaller ones. To score the size of drawn elements, the relative sizes on the sketch maps were considered. If the size of an element was in line with the size of its surrounding elements, it was accepted as a correct size depiction. Based on the average scores of all participants, the average shape score was 82.7 (N=54, MED= 83.3, SD= 12.2). Accordingly, experts drew map elements 7.8% better than novices did considering their size, and based on Mann-Whitney U test, the size scores, with U_size_= 244.5 and p= 0.040. The greatest difference occurred for settlements (14.3%) (Figure 10). A possible explanation could be that the depiction of settlements requires higherlevel generalization knowledge. Since individual buildings come together to form a settlement or village, aggregation is needed to define a group of buildings as a settlement. On the other hand, no significant gender difference emerged, according to Mann-Whitney U test (U_size_ = 283.5, p= 0.254).

**Figure 11.**
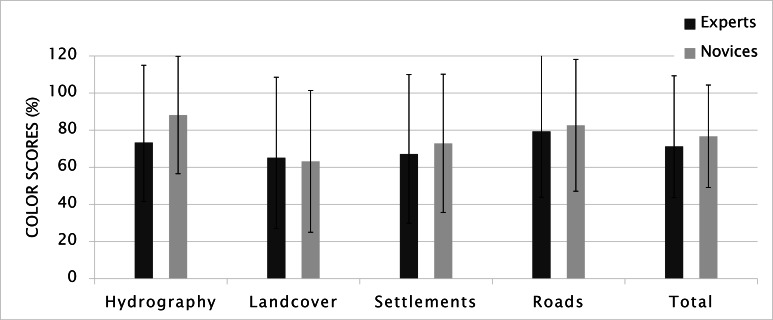
Color scores (Error bars indicate standard deviation).

During the drawing process, participants did not receive any information about using colors. However, the color palette embedded in MS Paint was available to all participants. Other than three novice and five expert participants who chose to use only black, the remaining participants delivered colored sketch maps. Our color assessment criteria regarded the color correspondence of an element drawn on the sketch map with the one on the original map. We also paid attention to whether the elements drawn in the same color represent the same category. Based on the average scores of all participants, the average color score was 75.0 (N=54, MED= 83.3, SD= 31.2). Novices depicted the map elements slightly better using corresponding colors. However, this surprising difference between novices and experts was not statistically significant regarding to Mann-Whitney U test (U_color_= 342.5 and p= 0.753). The greatest difference in performance was in hydrology (14.9%) (Figure 11). This result can be related to missing map elements on the sketch maps (since we assigned a score of zero to absent elements) or to the fact that some experts did not prefer to use color.

Although women were superior to men for the depiction of colors with 1.7% performance difference, no significant difference occurred among these two groups (U_color_= 342 and p= 0.934).

**Figure 12.**
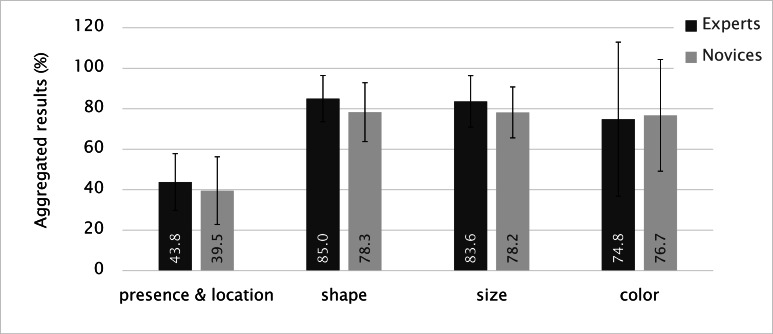
Summary of performances (Error bars indicate standard deviation).

Figure 12 shows the performances of experts and novices based on shape, size, color, and presence & location. We clearly see that the lowest overall performances for both groups occurred for presence & location. This result proves that drawing a map element in the correct location was more difficult than describing its shape, size, and color.

The sample size was not sufficient to study the differences of four groups; expert males (N= 11), expert females (N= 13), novice males (N= 24) and novice females (N= 7).

#### Aggregation presence & accuracy (1), shape (2), size (3) & color (4).

A Kolmogorov-Smirnov test was used to test for normality on the aggregated scores (D(54) = 0.126, p= 0.027) and the test results indicated that the data was not normally distributed. According to the aggregated analysis, the average score of experts was 71.8 (N= 24, MED= 76.8, SD= 19.2) with a minimum of 39.9 and a maximum of 92.8, whereas it was 68.2 (N= 30, MED= 68.8, SD= 11.1) with a minimum of 36.3 and a maximum of 92.2 for novices. The difference of 3.6% on expertise was not statistically significant regarding to Mann-Whitney U test (U= 254 and p= 0.065). The results implied that experts and novices showed no difference in map learning, unless the stimulus required specific map knowledge that only an expert possessed.

The average score of females was 73.2 (N=20, MED= 75.7, SD= 14.5) with a minimum of 39.9 and a maximum of 92.8, whereas it was 68.7 (N=34, MED= 70.3, SD= 2.2) with a minimum of 36.3 and a maximum of 92.2 for males. The difference among genders was not statistically significant regarding to Mann-Whitney U test (U= 264.5 and p= 0.146). Although it was not possible to make generalized assumptions or draw conclusions regarding to gender differences between experts and novices as explained earlier, the results showed that both expert and novice females were favored in their groups. Expert females were the most successful group overall with a score of 74.2. Novice females (69.9), then expert males (69.3) and lastly novice males (66.5) followed them.

### Eye Tracking

While studying the map, the average duration of the fixations was 230.0 ms (N= 24, MED= 230.8 ms, SD= 50.1 ms) for experts and 244.1 ms (N= 30, MED= 243.0 ms, SD= 48.4 ms) for novices. These values were 234.0 ms (N= 20, MED= 239.8 ms, SD= 56.3 ms) for females, and 240.1 ms (N= 34, MED= 232.8 ms, SD= 45.3 ms) for males. A Kolmogorov-Smirnov test was used to test for normality on the average duration of the fixations indicated that the data was normally distributed: D(54)= 0.082, p= 0.200.

The average duration of fixations for novices was slightly higher than it was for experts, whereas only slight differences emerged between the expert and novice groups and between females and males. However, according to two-way ANOVA, no significant difference was found (F(1,55)= 0.074, p= 0.787) between experts and novices, as well as between females and males (F (1,55)= 1.001, p= 0.322). Further, Cohen’s effect size value (d = 0.09) suggested that the effect was rather small for expertise (d= 0.123) and gender (d= -0.289).

The average number of fixations per second for the stimulus was 3.5 (N= 24, MED= 3.7, SD= 1.0) for experts and 3.6 (N= 30, MED= 3.6, SD= 0.5) for novices. These values were 3.4 (N= 20, MED= 3.4, SD= 1.1) for females, and 3.7 (N= 34, MED= 3.7, SD=0.5) for males. A Kolmogorov-Smirnov test was used to test for normality on the number of fixations per second indicated that the data did not fit normal distribution: D(54) = 0.145, p= 0.007.

The average number of fixation of novices and experts slightly differed, as well as it did for females and males. Regarding to Mann-Whitney U test, the differences emerged neither from expertise, nor from gender were statistically significant (U_expertise_= 338, p= 0.702; U_gender_= 254, p= 0.123).

**Figure 13.**
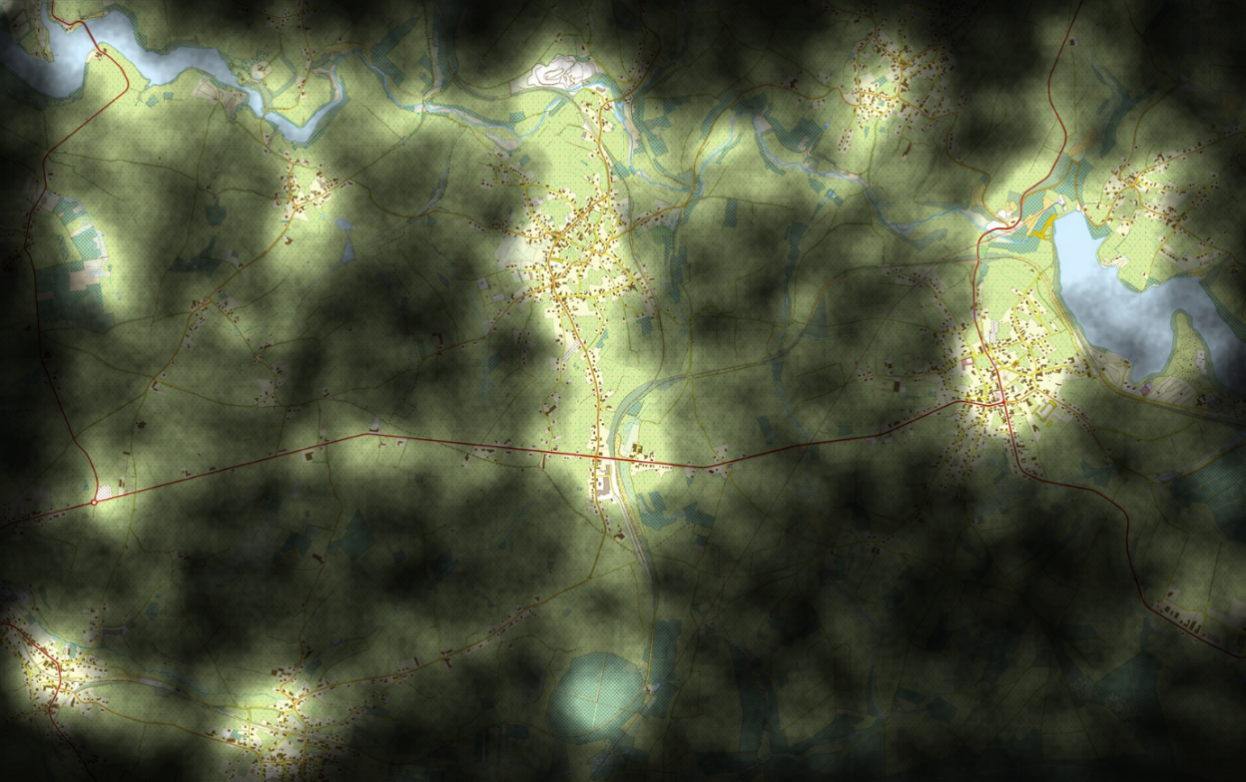
Focus map of all 54 participants.

**Figure 14.**
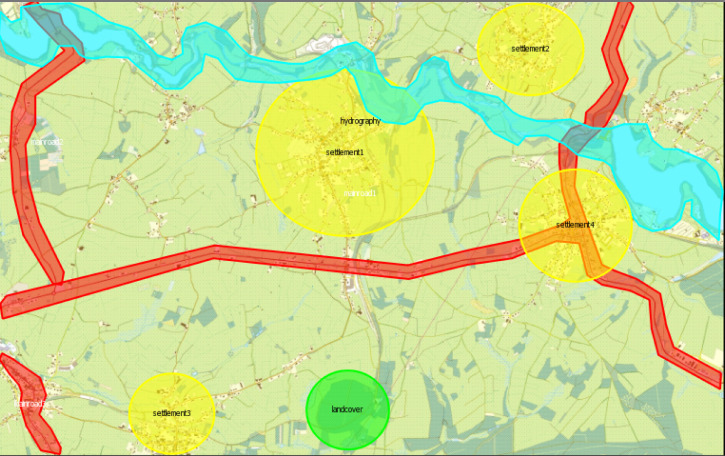
Selected AoIs

Having visually inspected, we observed that the gaze behaviors of all participants depicted in the focus map clearly reflect the main structural elements of the map stimulus (Figure 13). When visually interpreted, the focus map highlighted the main road construction, water bodies and large settlements belonging to the stimulus. The river located in the upper side of the map especially stood out. This result proves why the hydrography was the most remembered category with the highest score in drawing order. Furthermore, forests located on the bottom-right of the map look almost dark, which proves that the participants showed less interest in this part of the map. This finding supports the fact that the land-cover was the least drawn category (see results for drawing order) and also corresponds to what was registered by Ooms et al. [ 5 ]. Therefore, we could use the proposed AoI around the main structuring elements on the map. The AoIs considered for the further analysis include all three main roads and hydrographic elements, which are aggregated as a single object, four settlements, and one landcover object as depicted in Figure 14. Road 1 with the tilted Y-shape is located in the lower center of the map and forms the longest road feature. The largest settlement is the one located in the upper center of the map (Settlement 1), whereas another fundamental linear feature, the hydrography, covers the upper side of the map.

The AoIs considered for the further analysis include all three main roads and hydrographic elements, which are aggregated as a single object, four settlements, and one landcover object as depicted in Figure 14. Road 1 with the tilted Y-shape is located in the lower center of the map and forms the longest road feature. The largest settlement is the one located in the upper center of the map (Settlement 1), whereas another fundamental linear feature, the hydrography, covers the upper side of the map.

The time to the first fixation reflects that the larger objects and the objects located in the upper middle of the screen caught a participant’s attention earlier than the others did. Both experts and novices gazed at Settlement 1 first (350.7 ms for experts, 49.4 ms for novices), Road 1 second (3463.0 ms for experts, 3162.2 ms for novices) and Hydrography third (3821.1 ms for experts, 4455.2 ms for novices). The longest time to the first fixation was spent for the land-cover object (24976.8 ms for experts, 29863.9 ms for novices) that is located in the bottom-center of the map and has a relatively smaller size.

The dwell times of participants for all AoIs showed that there was similar behavior between experts and novices. The dwell times of experts were higher for Hydrography, whereas novices spent more time for Settlement 1. Both group spent less time for Roads 2 and 3, approximately 1/10 of what they spent for Hydrography and Settlement 1.

**Figure 15.**
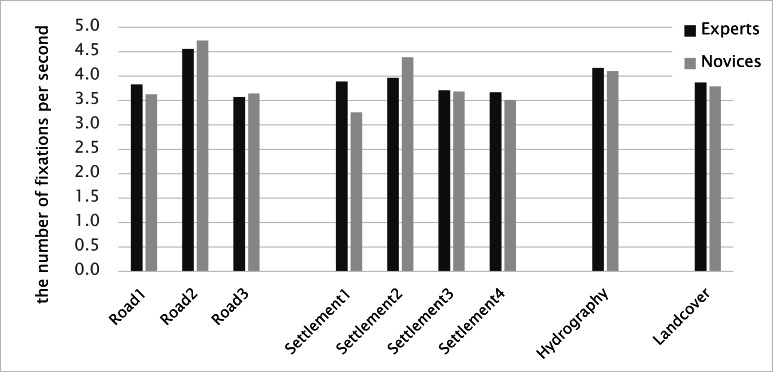
The number of fixations per second

On the other hand, the number of fixations within AoIs was slightly higher for experts. Hydrography received the highest fixation counts with 57.4 for experts and 46.5 for novices. The next highest numbers of fixations occurred for Settlement 1 and Road 1 (Figure 15). These map elements also resulted in longer dwell times. The fixation count was closely linked to the time a participant spent for a certain region (dwell time). Therefore, the number of fixations per second is a more objective measure to explore differences between experts and novices.

**Figure 16.**
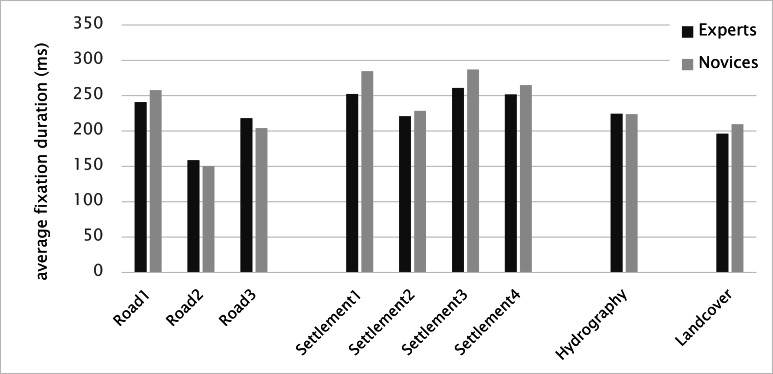
Average fixation duration (dark bars: experts, light bars novices)

The average fixation durations of participants were higher for all settlements (except Settlement 2) and Road 1 regardless of the expertise. Settlement 3 received the highest average fixation duration, whereas Road 2 received the lowest (Figure 17). Although both objects have relatively small sizes, participants seemed to have different reasons why they fixated on those objects for longer or shorter periods. The complexity of the object mostly resulted in higher fixation durations. In this case, the settlement was a more elaborate object compared to the road and required more processing time and thus, more cognitive load. Furthermore, our results proved that the fixation duration and the number of fixations were inversely proportional. The shorter the fixation duration was, the higher the number of fixations per second. For instance, Settlement 3 had the longest average fixation duration (287.0 ms, see Figure 16), while it received a lower number of fixations per second (3.7, see Figure 15) than the other objects did.

## Discussion

The results of the study are valid for a specific map stimulus representing only one specific area. However, the map, which was simplified only by removing altitude lines and labels, is a part of a map series covering the whole territory of Belgium. Therefore, the same trends could be observed on all these maps as they are based on the same symbology, although the generalization of results is limited. Although between- subjects design provided some potentially valuable insight, the outcomes may not apply for every condition. The performance of individuals is mainly influenced by the task and stimulus because the cognitive load can be manipulated by the complexity of the visual material and the difficulty of tasks. Therefore, if this study is extended by including other types of map stimulus and tasks, different results might be obtained.

The memory task explained in the paper required recalling the main structural elements of a screen map. This retrieval act involved WM-LTM transitions, such as retrieval of spatial information stored in WM through LTM or strategies for constructing hierarchy among map elements.

We regarded visual variables such as location, shape, size and color as though they were equally important for the drawing order which can be influenced by the use of visual variables. Besides other visual variables, color has long been recognized as a preattentive feature [ 32 ]. The order of drawing varied between participants, so that experts drew roads (depicted as red) in first place, whereas novices drew the elements hydrography (depicted as blue). Same situation applies for female and male participants, respectively.

In the original stimulus, roads were linear objects depicted in red, whereas hydrographic objects could be linear (rivers) or areal (water bodies) representations depicted in blue. Our retina includes light -sensitive cells named rods and cones. While rods mediate night vision, cones play role in photopic vision (during daylight) [ 69 ]. The spectral sensitivity of cones follows the order of the visual spectrum. Therefore, our eyes perceive the most in red wavelengths (500-760 nm) and the least on blue wavelengths (380-550 nm), and green wavelengths (430-673 nm) fall under the red range [ 70 ]. To the best of our knowledge, in map design, red tends to focus in the foreground; yellow and green, in the middle; and blue, in the background [ 71 ]. Thus, important objects or the ones to emphasize are shown in red, and blue is a good color for backgrounds. This feature could be the reason why the experts drew the red linear objects (roads) first. On the other hand, having drawn the hydrographic elements first, novices might have found areal objects as important or interesting and thus as memorable as linear objects. We can infer that size is as important as color for the retrieval of an object. Except for one participant, all novices drew water bodies on their sketch maps regardless of the order. Therefore, it is suggested that experts and novices use different strategies in spatial orientation, as well as females and males do. For instance, men tend to refer to environmental geometries or structuring elements, while women rely on landmarks [ 2, 72 ]. However, the common characteristic of the first drawn elements by all participants was that they both contained linear objects. This finding referring that the structuring elements guide spatial recognition is in line with what Edler, et al. [ 42 ] and Ooms et al. [ 6 ] found. Additionally, the hydrography category included lakes, which were areal representations. Starting with the areal elements instead of linear ones (or in our case, polygons (lakes) and lines (rivers) that were parts of a whole (hydrography)) proves that size of an object also plays an important role when recalling map information.

Based on the assessment of sketch maps considering the aggregated analysis of presence & location, shape, size, and color of drawn elements, we concluded that neither expertise, nor gender differences were influential on the retrieval of spatial information. Our findings related to gender differences corresponds to those by Lloyd & Steinke [ 73 ], Patton & Slocum [ 74 ], Beatty & Bruellman [ 75 ], [ 76 ], Lloyd & Bunch [ 77 ]) and Edler et al. [ 42 ]. On the other hand, our findings on the influence of expertise agree with the earlier research of Thorndyke & Stasz [ 1 ] who focused on experts’ and novices’ abilities to learn and remember information presented via maps. The fact that novices and experts did not differ in terms of how they learned and remembered map-related information could be explained by the general map knowledge that stepped in when both user groups observed a typical planimetric map stimulus. Hence, various levels of map experience may have resulted in modest differences [ 24 ]. The original map shown to participants was a simplified 1:10k topographic map and did not contain any familiar places (or names) to eliminate or minimize the degree of familiarity. Thus, both experts and novices observed the map for the first time, and we presumed that the maplikeness of the stimulus had a great influence on their map learning (study and recall) process. However, the later work (e.g. [ 6, 24, 78 ]) failed to replicate Thorndyke & Stasz’s [ 1 ] findings. Instead, they found that experts performed better in recalling schemas in a richer and more detailed fashion. Although our results present that experts and novices do not differ in terms of the amount of information they recall, the learning/recalling strategies of experts and novices may differ. The drawing order results could be evidence that they might use different approaches.

In addition to the maplikeness and the simplicity of the map, the task to be executed was influential on performance. It is important to remember that if the task required domain-specific knowledge about geography or related areas, experienced users would perform better compared to novices [ 1, 24 ]. Although individual factors other than expertise and gender might have affected the results, the sample size was not sufficient to draw conclusions regarding ethnicity or native language.

While encoding spatial information through maps, structuring elements (e.g. topographic details and grid lines) lead attentional shifts towards “to-be-learned object locations” which improve memory performance. The fact that the first fixation is influenced by experimental manipulations can be seen during recognition and it suggests that the structuring elements are involved in cognitive map production [ 63 ]. Therefore, eye tracking metrics provided valuable insight on how mental representations formed. In this context, average fixation duration and the number of fixations per second revealed that there was no significant difference between the expert and novice groups, as well as between men and women. Although this outcome was different from what was found by Ooms et al. [ 5 ], it supports our results obtained by digital sketch map assessment.

In addition, the eye tracking metrics (time to first fixation, dwell time, fixation count, the number of fixations per second, average fixation duration) for selected AoIs were explored. The time to first fixation statistics showed that larger AoIs were gazed at earliest and the dwell times for such objects were much longer compared to those for other AoIs. As expected, the majority of participants drew these map elements on their sketch maps. On the other hand, most participants paid less attention (late first fixation and less dwell time) to the relatively small linear (i.e. roads) and areal features (i.e. land cover) within the specified AoIs. However, when comparing the presence and accuracy scores of drawn elements, both groups mostly drew small roads on their sketch maps but not land-cover features. We could infer from this result that the linear features were easier to learn and remember, although the viewer did not pay much attention. Additionally, our results supported the fact that shorter fixation durations resulted in higher numbers of fixations per second. Consequently, longer average fixation durations for a specific AoI indicated that the chances were higher to remember that object. This finding corresponded to the number of objects depicted on the sketch maps; the objects that were absent on the sketch map received the shortest fixation durations during the study phase. However, longer fixation durations may also indicate participants’ difficulty to recognize the information in the observed visual scene.

Although it was beyond the scope of this study, the sequence of visited AoIs can be further explored to analyze how the map elements within specified AoIs are associated to form a sketch map. The sequential order of included elements may vary among individuals who draw sketch maps of the same map stimulus and sequence analysis can provide more insightful outcomes related to how map users encode structure, learn, remember and later use the spatial information presented via maps (e.g. [ 67 ]). Furthermore, the similarity between sequences can be studied by quantifying and comparing scanpath behaviors of individuals. Scanpath analysis promises rich information regarding to spatial and temporal characteristics of eye movements and contributes to understanding individual differences in a more systematic way (e.g. [ 79, 80 ]).

## Conclusion

This study utilizes digital sketch maps to understand the cognitive abilities and limitations of map users during a memory task via drawing. On one hand, we assessed the quality of sketch maps based on the drawn elements (e.g. the influence of visual variables), which we predicted would reflect the performances of different user groups and might reveal significant insights about their cognitive processes and strategies of retrieving spatial information. On the other hand, we integrated ET statistics to quantify the cognitive processes to advance time-related, gaze activity-related (especially fixations) analyses. We also derived the order in which the sketched objects were drawn from the ET data. The order of drawing offered significant insight into the hierarchical construction of cognitive maps and might have unveiled the differences in the retrieval strategies of experts and novices, if there were any.

Instead of traditionally used pen and paper method, we collected sketch maps digitally to be able to match them with the corresponding eye tracking metrics. Therefore, ET and sketch map were considered as complementary user testing methods providing detailed insight into user behaviors. No significant differences emerged between experts and novices, as well as females and males based on sketch map analyses, and this result was also confirmed by a number of ET statistics. This finding arose from a user experiment that considered a simplified static map for a memory task related to the map elements. However, this research can be extended by considering more rapidly evolving cartographic stimuli (3D visualizations, interactive displays, mobile maps, etc.) and tasks that require different levels of expertise to achieve a better understanding of map users. The more we understand the cognitive limits and abilities of map users, the more we become able to create effective cartographic products.

### Ethics and Conflict of Interest

The authors declare that the contents of the article are in agreement with the ethics described in http://biblio.unibe.ch/portale/elibrary/BOP/jemr/ethics.html and that there is no conflict of interest regarding the publication of this paper.

### Acknowledgements

This research was supported by the The Scientific and Technological Council of TURKEY (TUBITAK) within 2214-A under Grant 1059B141600039 for Merve Keskin.
